# CRISPR-Cas9 Mediated Labelling Allows for Single Molecule Imaging and Resolution

**DOI:** 10.1038/s41598-017-08493-x

**Published:** 2017-08-16

**Authors:** Abdullah O. Khan, Victoria A. Simms, Jeremy A. Pike, Steven G. Thomas, Neil V. Morgan

**Affiliations:** 1 Institute of Cardiovascular Sciences, College of Medical and Dental Sciences, University of Birmingham, Birmingham, UK; 2 Centre of Membrane Proteins and Receptors (COMPARE), Universities of Birmingham and Nottingham, Midlands, UK

## Abstract

Single molecule imaging approaches like dSTORM and PALM resolve structures at 10–20 nm, and allow for unique insights into protein stoichiometry and spatial relationships. However, key obstacles remain in developing highly accurate quantitative single molecule approaches. The genomic tagging of PALM fluorophores through CRISPR-Cas9 offers an excellent opportunity for generating stable cell lines expressing a defined single molecule probe at endogenous levels, without the biological disruption and variability inherent to transfection. A fundamental question is whether these comparatively low levels of expression can successfully satisfy the stringent labelling demands of super-resolution SMLM. Here we apply CRISPR-Cas9 gene editing to tag a cytoskeletal protein (α-tubulin) and demonstrate a relationship between expression level and the subsequent quality of PALM imaging, and that spatial resolutions comparable to dSTORM can be achieved with CRISPR-PALM. Our approach shows a relationship between choice of tag and the total expression of labelled protein, which has important implications for the development of future PALM tags. CRISPR-PALM allows for nanoscopic spatial resolution and the unique quantitative benefits of single molecule localization microscopy through endogenous expression, as well as the capacity for super-resolved live cell imaging.

## Introduction

Single Molecule Localization Microscopy (SMLM) offers the highest spatial resolution (approximately 10–20 nm) of the many super-resolved light microscopy techniques currently available^1, 2, 4–7^. SMLM approaches, of which STORM and PALM remain eminent techniques, also offer unique quantitative benefits as they rely on localizing individual molecules, a principle which allows for an unprecedented level of detail in the study of protein-protein interactions in terms of spatial distribution, stoichiometry, and clustering^2, 3^.

STORM (STochastic Optical Reconstruction Microscopy) was developed as one of the first SMLM techniques, successfully combining the stochastic activation of fluorophores with computational reconstruction for nanoscopic imaging, originally through the use of a Cy5-Cy3 pair to achieve the cyclical switching of subsets of emitters^1, 4–11^. The dSTORM (direct Stochastic Optical Reconstruction Microscopy) approach relies on the use of single labels on fixed samples, allowing for the convenient labelling of a sample, whilst also exploiting the high photon counts of specific fluorophores (e.g. Alexa-647, Atto488) attached to commercial secondary antibodies, thus allowing for highly precise single molecule localizations^2, 3, 5, 7, 12^. While extremely popular and effective due to its relative simplicity, dSTORM is limited to fixed samples which precludes the option of live cell imaging, and similarly relies on the use of high affinity antibodies to minimize background and achieve a sufficiently high labelling density. While the theoretical limit for the localization precision of individual molecules is dependent on the photon count, the effective resolution of a reconstructed SMLM image is also determined by factors such as sample and stage movement, and crucially the labelling density of the protein of interest; the higher the labelling density and sampling rate, the higher the achievable resolution to a point defined by the Niquist-criterion^13–16^.

A number of alternative dSTORM approaches have been developed to further refine the technique. These include the use of smaller tags, as the size of antibody labels (10–15 nm) can have a significant impact on spatial measurements at the nanoscopic level^17^. The use of nanobodies (approximately 5 nm in size) for example, has been used effectively to resolve the hollow structure of microtubules^17^. Similarly, a number of chemical tags allow for dSTORM while overcoming issues with antibody affinity, including TMP, SNAP, and HALO tags, each of which offers distinct advantages and disadvantages^18–21^. However a key quantitative issue remains amongst these approaches, namely repeated blinking events from each labelled molecule^12, 22^. The problem of multi-emitters is inherent to the stochastic activation of fluorophores, and remains a significant area of focus and interest in the field of SMLM, with efforts directed at computationally addressing the problem by defining specific fluorophore stoichiometries and switching kinetics^15, 23^.

PALM (PhotoActivated Localization Microscopy) utilizes photoswitching and photoactivatable fluorophores (e.g. rsDronpa, rsFastLime, PAMCherry1, mEos) to achieve the ‘blinking’ data sets required for single molecule imaging^4, 8, 9^. This approach offers a number of distinct advantages, for example, a number of PALM fluorophores address the multi-emitter issue (e.g. mEos) such that that each detectable emission can be more accurately assigned to a single molecule^2, 3^. PALM also offers the option of live cell imaging as the relevant tags are traditionally introduced through transient transfection of an appropriately labelled fusion protein^24–27^. PALM tags offer defined switching kinetics, with many irreversible photoswitching tags which upon activation and bleaching do not reversibly switch, allowing for more accurate quantification^23, 28, 29^.

Unfortunately PALM also suffers from a number of drawbacks, most significant of which is that engineered photoswitching tags typically demonstrate poorer photon counts and contrast ratios than labels used by dSTORM. This affects localization precision and the quality of rendered super-resolution images^24, 27, 30, 31^.

Whilst the over-expression of fusion proteins can help obtain the labelling density required for high effective resolution, this approach often results in aberrant protein expressions with functional consequences, which, alongside the experimental variation inherent to transfections are key biological limitations of the technique^10, 11^.

The targeted insertion of fluorescent tags into specific chromosomal loci has been achieved in bacteria and applied for PALM through endogenous expression, offering a distinct advantage over the traditional approach of transient transfection and allowing for fixed and live cell PALM^32–35^. Unfortunately until recent years, efficient techniques for the targeted insertion of fluorescent tags into eukaryotic cells have been lacking.

The recent introduction of CRISPR systems as highly efficient, targeted, and inexpensive methods for gene editing in mammalian cells has significant implications in the field of super-resolved imaging^2, 10, 12, 13^. Firstly, genetically tagging a protein of interest will result in the generation of stable expression patterns under normal cellular regulation and control, thus resulting in healthier expression profiles which reduces the functional impact of protein tagging^36, 37^. Moreover, removing the variation inherent in plasmid transfection allows for a stable platform for biological reproducibility.

By genetically expressing tags of interest under the control of endogenous promoters, a direct relationship between cellular expression and fluorescent signal can be established. Such an approach has vast potential for SMLM, where single molecules are detected and counted (particularly in PALM), and where there is a direct and quantifiable relationship between the genomic expression of a tagged protein and its super-resolved detection. Thus, a CRISPR mediated PALM approach presents an unprecedented opportunity to quantitatively answer biological questions at the single molecule level.

To date two studies have explored the effect of endogenous protein tagging on super-resolved imaging^2, 10^. Of these, one has adopted the SMLM approach (PALM) for the labelling of DNA Pol/II. However a key issue in SMLM imaging is whether or not it is possible to sufficiently label a highly abundant protein in a manner which satisfies the stringent sampling required for high spatial resolutions. Thus far, this question remains unanswered.

Similarly, wider questions remain with regards to the effect of applying CRISPR to knock-in genomically expressed tags. We are unaware of any work which quantifies the effect of such a tag on the total expression of fusion proteins compared to wildtype, thus, it remains unclear to what extent regulatory mechanisms affect the expression of tagged genes in CRISPR edited cells. Similarly key properties of fluorescent proteins are likely to have an impact on the expression of endogenously expressed fusion proteins, including maturation rates and the tendency to oligomerize^27, 38^. Key aspects of fluorophore design are likely to have a significant effect on the quality of CRISPR-PALM data sets, and impact both the resolution achieved and the accuracy of any resulting single-molecule quantification.

To date CRISPR-Cas9 mediated tagging has been shown to ameliorate over-expression issues, resulting in the expression of fusion proteins while retaining healthy cell morphologies and functionality^10, 14^. In this study we investigate the effect of labelling the α-tubulin gene *TubA1B* with mEGFP and the photo-switching PALM tag mEos 3.2 in three different cell lines, Hek293T, Hel 92.1.7, and A549s. *TubA1B* is expressed across a range of cell types, making it an ideal target for the study of tubulin. We show that the expression level of labelled α-tubulin varies across cell types and fluorophores, with mEGFP consistently more highly expressed than mEos 3.2.

We demonstrate that at high enough expression levels, such as those exhibited by Hel 92.1.7 cells, a spatial resolution comparable to dSTORM is achievable, while also allowing for super-resolved live cell imaging and convenient multiplexing with dSTORM. These findings are significant for the field in that they indicate a need for tags optimized for expression in mammalian cells to achieve optimal PALM, however they also highlight the potential for high quality SMLM presented by endogenously expressed PALM fluorophores. While the benefits of CRISPR-PALM lie in accurate single molecule quantitation of genomically regulated proteins without the biological variance and disruption caused by transfection, achieving high effective resolutions at these expression levels is a significant advantage.

## Results

### CRISPR/Cas9 labelling of *TubA1B* with mEGFP and mEos 3.2

Three cell lines expressing *TubA1B* with distinctive morphologies, lineages, and expression levels were selected for gene editing (Hel 92.1.7, A549, and Hek293T). *TubA1B* is widely expressed in human cell lines as well as induced Pluripotent Stem Cells (iPSCs), making it an excellent candidate for comparing expression levels and the performance of CRISPR-PALM across different cell types^39–41^. mEGFP was chosen as a stable fluorescent protein optimized for mammalian expression in comparison with mEos 3.2 which, while an excellent PALM protein with a high photon count, has demonstrated a tendency to oligomerize and have a comparatively lower maturation rate^27, 30, 38^.

**Figure 1:**
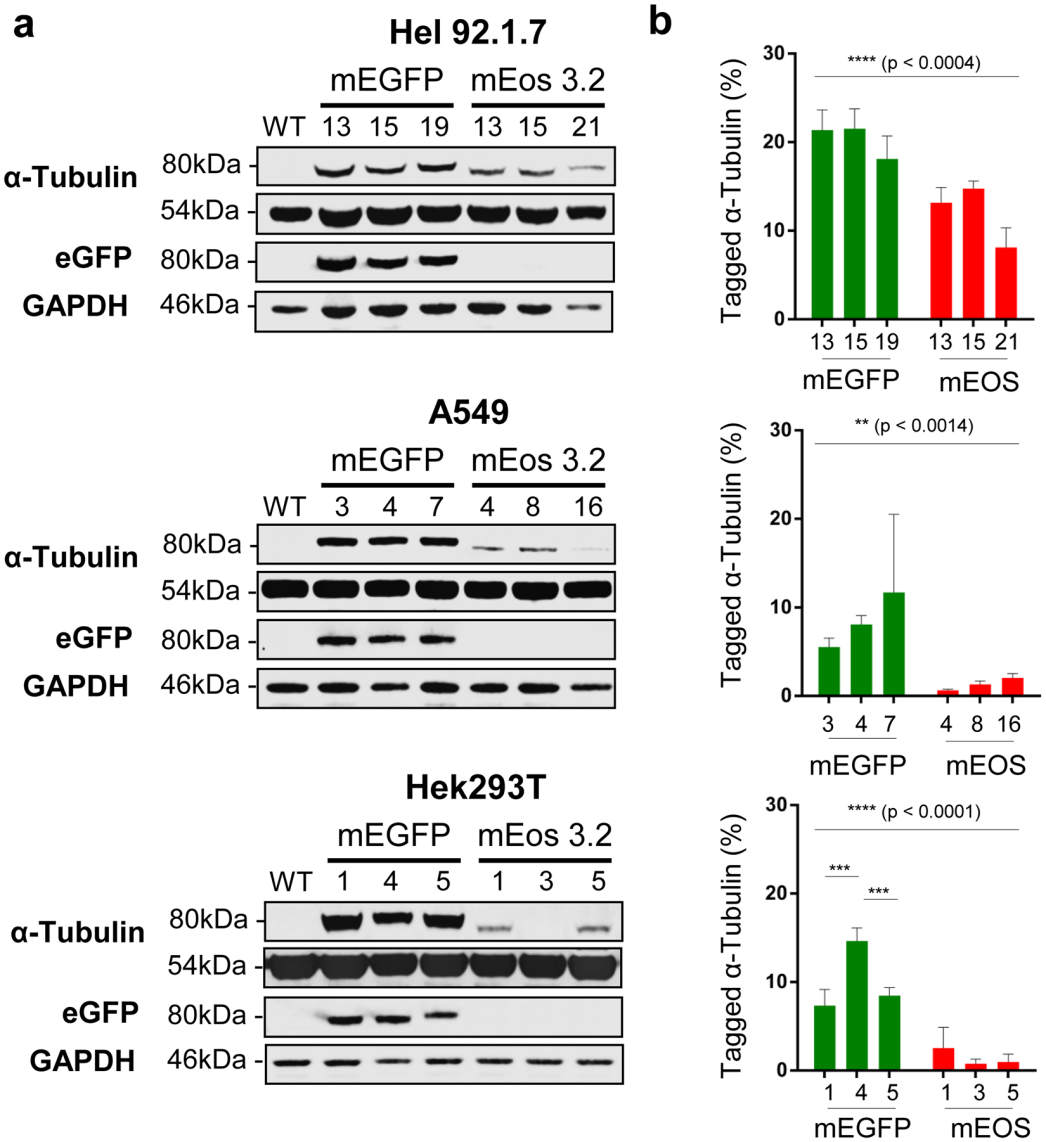
Western blotting and quantitation of tagged α-tubulin mEGFP and mEos 3.2 clones. (**a**) All three cell lines express a tagged α-tubulin at the predicted molecular weight of approximately 80 kDa. Use of an anti-GFP antibody detects these bands in eGFP expressing clones. (**b**) All three cell lines demonstrate a significant difference in expression between eGFP and mEos 3.2 tagged TubA1B (****p < 0.0004 in Hel 92.1.7, **p < 0.0014 in A549, and ****(p < 0.0001), with little clone to clone variation in the samples tested. Statistical testing performed through a Two-Way ANOVA with multiple comparisons (Tukey). Full length western blots and quantitation of endogenous TubA1B expression levels are supplied in Supplementary Fig. 6 and 7.

Guide RNAs were generated targeting the N-terminal region of *TubA1B* ^15^. Similarly homology arms flanking the *TubA1B* target sites and appropriate mEGFP and mEos 3.2 donors (Supplementary Fig. 1) were designed and cloned to generate donor plasmids. Cells were transfected with the appropriate guide RNAs and donors before fluorescent cell sorting (FACS) (Supplementary Fig. 2) to generate single cell colonies. These cells were grown, screened for the presence of an insert at the target genomic locus (Supplementary Fig. 3), before Western blotting to determine the total expression of tagged protein (Fig. 1). Single clones for each cell type and tag were selected on the basis of expression level and subsequently taken forward for imaging experiments.

A significant difference in total tagged tubulin expression was noted between mEGFP and mEos 3.2 cells across all three cell types (Fig. 1). A comprehensive review of fluorescent proteins has recently shown that mEGFP remains the most highly monomeric tag available, and despite improvements in mEos that have steadily improved this particular quality, it remains markedly more oligomeric^8, 9, 16, 17^. This reduced oligomerization, combined with the improved maturation rates and codon optimization demonstrated by mEGFP is likely to account for its relatively higher expression level across cell lines when compared to mEos 3.2.

Hel 92.1.7 cells express both *TubA1B*-mEGFP and *TubA1B-*mEos 3.2 more highly than both Hek293T and A549 cells (Fig. 1). This variation across cell type is most dramatic in mEos 3.2 tagged *TubA1B* clones, where Hel 92.1.7 express approximately 15% tagged tubulin compared to as little as 1–2% in Hek293T and A549s (Fig. 1).

The majority of clones generated were heterozygous for their relevant insertion (Supplementary Fig. 3), however while Hel 92.1.7 *TubA1B*-mEos 3.2 clones 13 and 15 demonstrate a loss of wild type band, there is no significant difference in expression compared to Hel 92.1.7 *TubA1B*-mEos 3.2 clone 21 (which retains the wild type band – Fig. 1). This observation is surprising, however consistent with Ratz *et al*.’s finding that vimentin knock-ins were heterozygous compared to other targeted genes, which demonstrated a mix of higher insertion efficiencies. Interestingly, even in Hel 92.1.7 cells which most highly express tagged α-tubulin, expression does not exceed ~20% for mEGFP and ~15% for mEos 3.2 (Fig. 1b), suggesting that other isotypes of α-tubulin may be expressed in these cells, or that fluorescent knock-in may cause partial silencing of the gene in question.

### CRISPR-PALM Imaging of mEos 3.2 Knock-Ins

**Figure 2:**
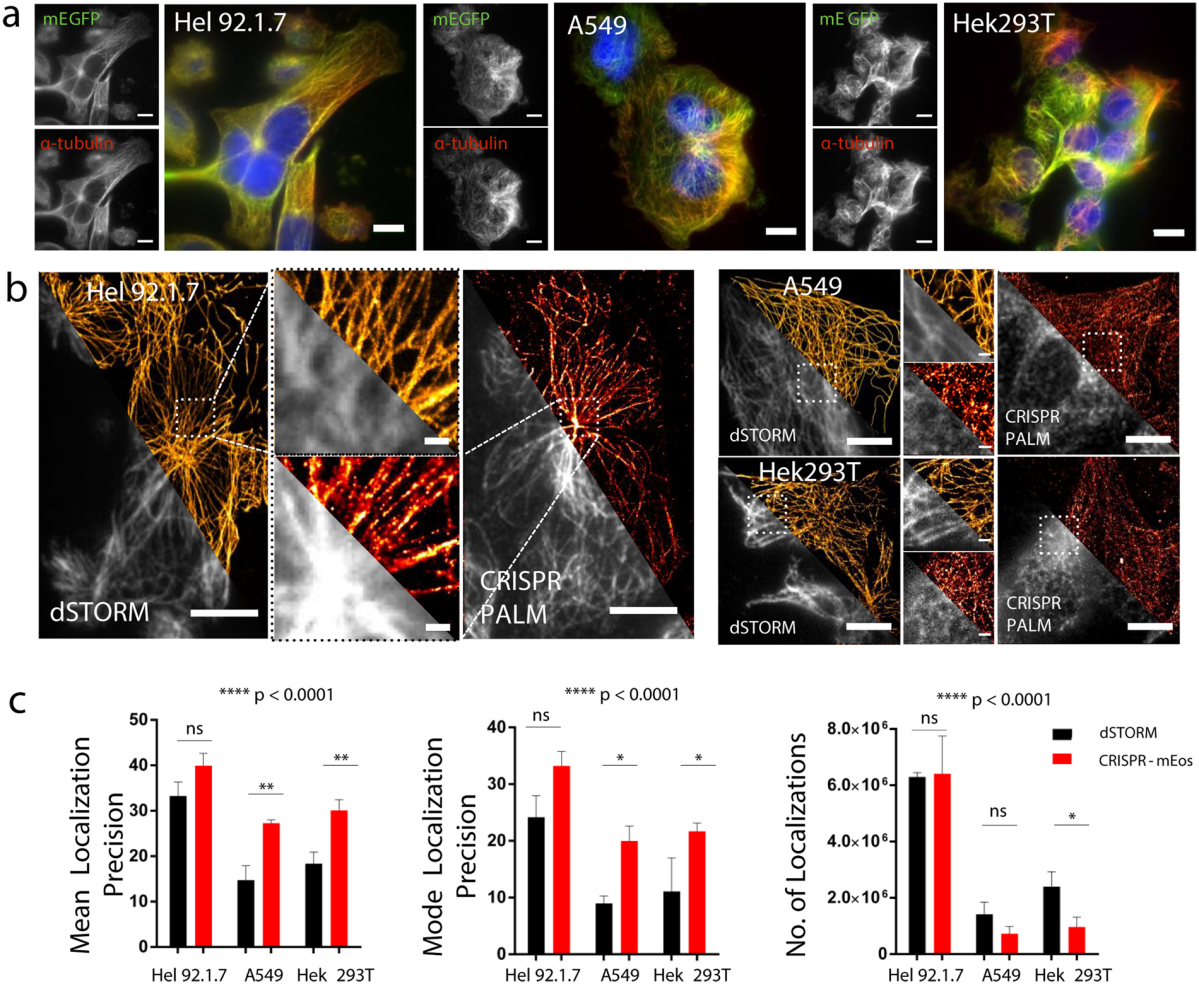
Labelling of *TubA1B* gene through CRISPR-Cas9 genome editing in 3 cell lines. (**a**) mEGFP tagging of *TubA1B* was compared to antibody based labelling in Hel 92.1.7, A549 and Hek293T cells. In all cases, gene editing results in the expression of an mEGFP α-tubulin fusion protein under endogenous control mechanisms (10 μm scale). (**b**) mEos 3.2 tagged *TubA1B* allows for the endogenous expression of a photoswitchable tag, and thus PALM imaging. Resolution is similar to that observed for dSTORM images in Hel 92.1.7 cells, and is dependent on the expression level of the mEos 3.2 fusion (**c**) Detection metrics from ThunderSTORM reconstructions evidence no significant difference between mEos 3.2 imaging in Hel 92.1.7 cells and dSTORM, while the lower expression level in A549 and Hek293T cells results in significantly reduced localization precisions (xy uncertainties) and number of localizations. Bars represent mean ± SD or Mode ± SD. (n = 3). Data were analysed using a 2-way ANOVA with multiple comparisons (Tukey’s). (10 μm scale in whole cell images, 1μm scale in cropped fields). Full sized images are supplied in Supplementary Fig. 4.

While the relatively low expression of *TubA1B*-mEGFP in Hek 293T and A549 cells still allows for the diffraction limited imaging of tubulin (Fig. 2a)^18^, the expression level plays a key role in labelling density and hence sampling rate and resolution. While mEos switching does allow some improvement in resolution in Hek 293T and A549 cells, microtubules observed in these samples are not as clearly resolved in continuous fibers as in their dSTORM counterparts (Fig. 2b). Conversely, the higher expression level observed in Hel 92.1.7 cells results in clear, continuous filamentous structures comparable to equivalent dSTORM images (Fig. 2b). These findings are corroborated by mean and modal localization precision and number of localizations yielded by the image reconstructions (Fig. 2c)^18^. While localization precision between dSTORM and CRISPR tagged samples are significantly different in poorly expressing cells like Hek293T and A549, Hel 92.1.7 cells show no significant difference when compared to dSTORM despite a mild decrease in expression.

**Figure 3:**
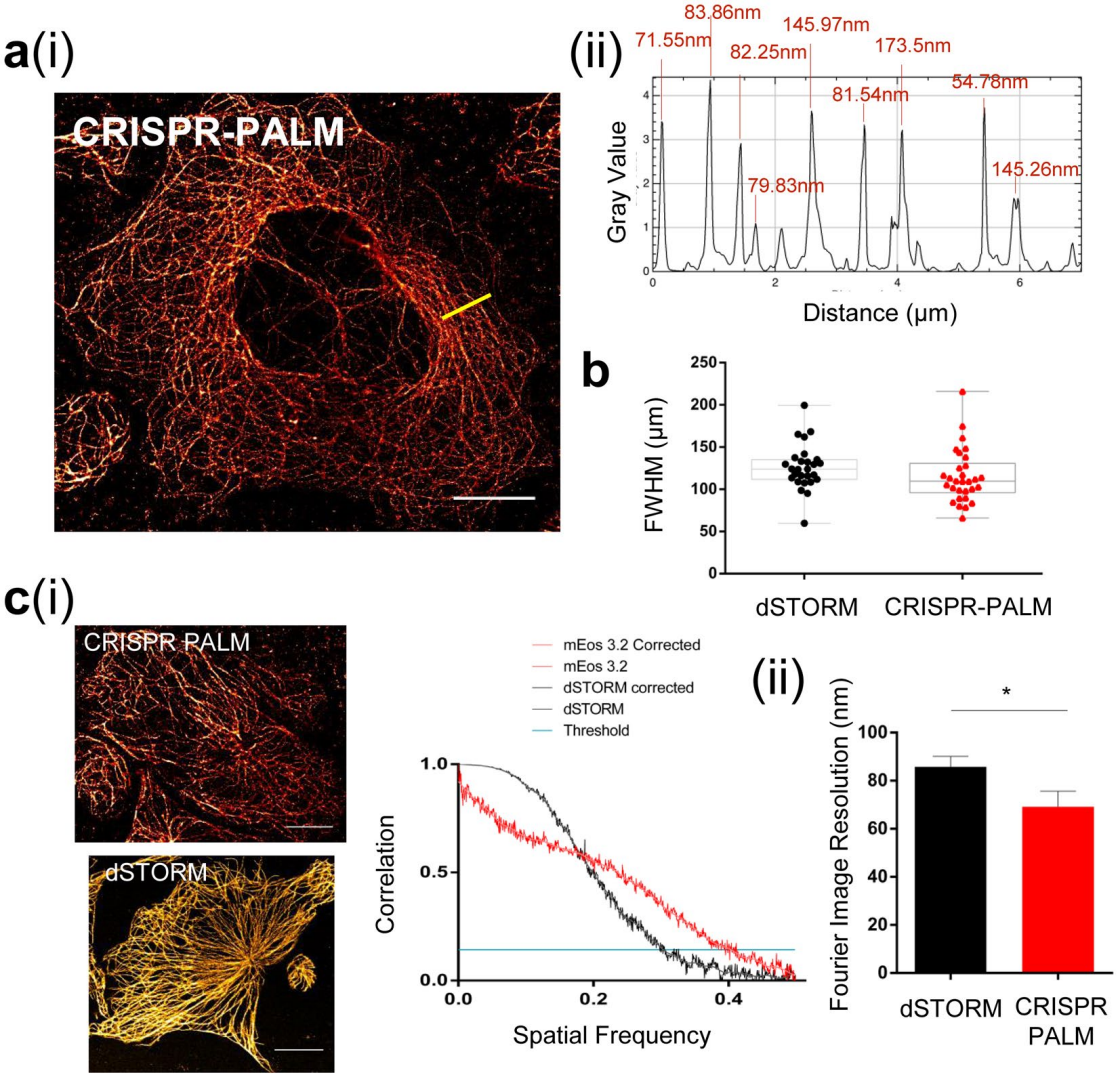
Genomic expression of a tagged *TubA1B* allows for SMLM equivalent to dSTORM images. (**a**) (i) PALM imaging of CRISPR mEos 3.2 results in clean, consistent super-resolved tubulin fibers despite the labelling density required for high quality cytoskeletal imaging. (ii) shows an example FWHM line profile for the yellow line in (a) (i). (**b**) Median Full width half maxima (FWHM) of line profiles across multiple cells shows that mEos 3.2 FWHM in Hel 92.1.7 cells is not significantly different when compared to dSTORM (median 123.91 (dSTORM) and 109.713 (mEos 3.2)) (**c**) (i) Applying the Fourier Ring Correlation method also reveals Fourier Image Resolution (FIRE) values in *xy* of 40 nm for mEos 3.2 and 72 nm for dSTORM representative figures. FRC plots for the representative images are shown from which is calculated the FIRE value. (ii) When measured across all data sets, a statistically significant improvement in FIRE is observed (*p = 0.0447). FWHM data generated from line profiles in Fiji, with measurements obtained from multi-peak analysis in Igor Pro. Box and whisker plot showing median with min and max values. FIRE values and FRC curves are calculated using FRC plug-in (Fiji), with an unpaired t-test used to test for significance. (10 μm scale).

No significant difference was observed in the FWHM of microtubules measured across a number of cells from different replicates when compared to dSTORM (Fig. 3a(i-ii),b). We also compared CRISPR mEos images with dSTORM in Hel 92.1.7 using the Fourier Ring Correlation (FRC) method to establish the resolutions within these images. The FRC method is used to calculate the Fourier Image REsolution (FIRE), a measure of effective resolution which accounts for uncertainty and labelling density, which are both critical considerations when determining the efficacy of a nanoscopic technique, and particularly when establishing the effect of endogenous expression levels on PALM imaging^20, 21^.

We show comparable FIRE values (Fig. 3(ci)) between these imaging modalities, which alongside the FWHM and localization metrics, convincingly demonstrate that CRISPR-PALM can achieve resolutions comparable to dSTORM, even when imaging a cytoskeletal structure which demands high labelling density to achieve true nanoscopic imaging.

These findings directly correlate detection and single molecule resolution in CRISPR tagged cells to protein expression level, as these resolutions are achievable in Hel 92.1.7 clones which demonstrate the highest expression of mEos 3.2 tagged α-tubulin. Interestingly, a statistically significant improvement in FIRE is observed (* p = 0.0447 Fig. 3(c(ii)), which is likely a result of more consistent fluorescent tagging of α-tubulin in cells genomically expressing mEos 3.2.

### Application of mEos 3.2 Tagged Cells for Live Cell Imaging and Multi-Colour SMLM

**Figure 4:**
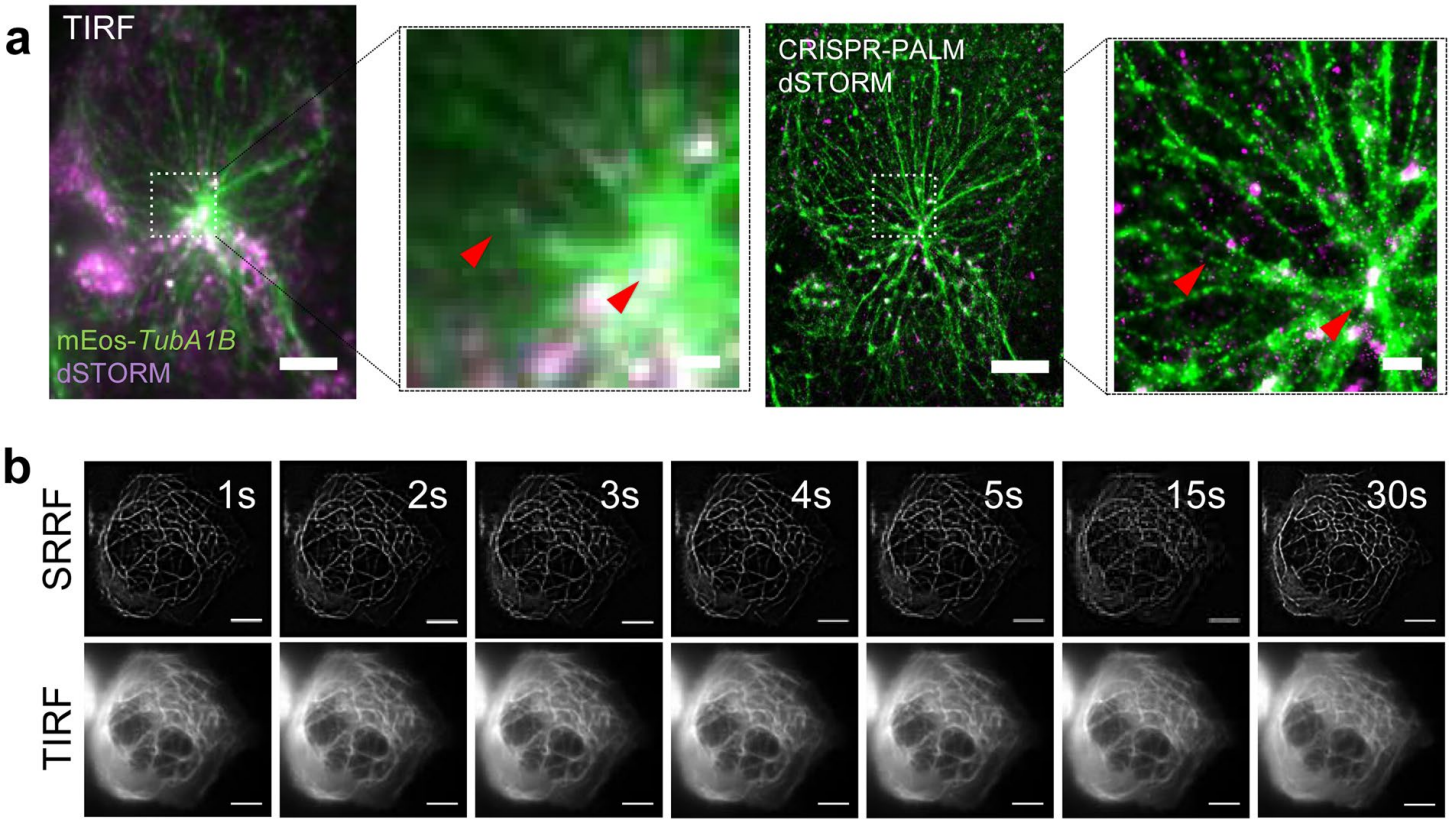
CRISPR-Cas9 mediated PALM is a powerful biological tool for probing protein interactions and live cell imaging. (**a**) mEos 3.2 tagging of the *TubA1B* gene allows for convenient multiplexing with high quality antibody probes for multi-colour dSTORM/PALM using the highest performing secondary, Alexa 647. TTLL10, a tubulin ligase is tagged with Alexa 647 and imaged in a Hel 92.1.7 mEos 3.2-*TubA1B* clone, providing super-resolved imaging of two proteins which are spatially distinct with resolution in the 10 s of nanometers, as indicated by red arrows. (**b**) The switching properties of mEos 3.2 can be further exploited to enhance sub-pixel radiality needed for high quality SRRF (super-resolved radial fluctuation) imaging, and thus for super-resolved imaging at high temporal resolution with minimal phototoxicity. The lower panel show averaged images from 50 frames of a TIRF image stream and the corresponding SRRF image. (10 μm scale in whole cell images, 1 μm scale in cropped fields).

A key benefit of SMLM is its ability to establish spatial and quantitative protein-protein interaction, and while multi-colour STORM and PALM have been successfully shown, there remain practical obstacles in establishing routine multicolour SMLM. Different antibody labels for example have distinct photochemical properties, and perform optimally in different buffers^31^. Similarly, multiplexing PALM fluorophores offers similar challenges, with issues in spectral compatibility and fluorophore performance^27^. CRISPR-PALM offers a simple alternative by combining the imaging of mEos 3.2 with a dSTORM label of choice (Fig. 4a).

Live cell super-resolution microscopy has made significant use of the non-linearity introduced by photoswitching fluorophores and their subsequent ‘blinking’ data sets. A number of modalities are available which exploit these properties, including 3B, SOFI, NL-PA SIM, and more recently SRRF^42–47^. We show that CRISPR tagged mEos 3.2 Hel 92.1.7 cells are suitable for performing rapid SRRF imaging, generating sub-diffraction images at sub-second temporal resolution (Fig. 4b, Supplementary Fig. 5 Supplementary Videos S1, S2). Importantly, these cells can be imaged through any one of a number of such approaches. As the properties of photoswitchable/activatable tags are increasingly applied in innovative super-resolution imaging modalities, CRISPR mediated endogenous expression is a powerful means of comparing these approaches in a stable system without the variability inherent to transient transfection

## Discussion

In this work we show that sufficient labelling density for high quality SMLM imaging can be attained through CRISPR tagging of a highly expressed cytoskeletal protein, and that an important consideration is expression level in the target cell type. Where an appropriately high level of expression is reached, as demonstrated here by Hel 92.1.7 cells expressing *TubA1B-*mEos, the resulting spatial resolution can be compared to the nanoscopic resolution achieved by dSTORM. Endogenous expression of mEos 3.2 tagged cells generates a stable biological model from which a number of imaging strategies can be evaluated. As new imaging approaches continually emerge, robust and reproducible biological systems for testing and comparison become increasingly important – as such genomic expression of tags of interest can be an invaluable asset. This is particularly true in SMLM and its quantitative applications, where single molecule detection of a genomically encoded tagged protein can allow for the accurate study of endogenous protein stoichiometries and spatial relationships.

The variation observed in the expression level of mEos 3.2 and mEGFP tagged tubulin implies that an improved understanding of how genome editing affects the expression of labelled proteins, as well as how key properties of these labels affect that expression, is needed to achieve sufficient sampling for nanoscopic resolution and accurate quantification. Factors like maturation rates and monomericity are likely to be key considerations in generating effective SMLM tags for endogenous expression.

Future work will aim to establish how the monomeric properties and maturation rates of PALM tags can improve total expression, and therefore improve the quality of CRISPR-PALM models. Once an improved understanding of these key considerations has been achieved, extending this approach to other genes will be a critical step in establishing CRISPR-PALM approaches as a unique and highly effective method of quantifiable single molecule imaging.

## Methods

### Cell Culture and Transfection

Human Embryonic Kidney 293T (HEK293T) and Human Alveolar Basal Carcinoma (A549) cell lines were cultured in complete Dulbecco’s Modified Eagle’s Medium (DMEM) with 10% Foetal Bovine Serum (FBS) supplemented with 1% Penicillin/Streptomycin (P/S) and 2mM L-Glutamine. Human Erythroleukaemia 92.1.7 (Hel 92.1.7) cells were cultured in Roswell Park Memorial Institute (RPMI) 1640 medium with 10% FBS, 1% P/S, and 2mM L-Glutamine. Cells were maintained in a 37 °C incubator with 5% CO_2_.

Transfections were performed on HEK293T and A549 cells using Lipofectamine 3000 (Thermo Scientific). Cells were seeded 24 hours prior to treatment at a density of 1 × 10^5^ on plastic bottomed 12 well plates (Thermo Scientific). Immediately before transfection, cells were washed twice with sterile Phosphate Buffered Saline (PBS) and incubated in antibiotic free complete medium for one hour. Lipofectamine 3000 and DNA mixes were prepared as described by the manufacturer. Plasmid DNA was prepared to a total of 1 µg per well, with equimolar ratios of Cas9-RFP (Sigma), BPK1520 guide RNA expressing plasmid (BPK1520 was a gift from Keith Joung (Addgene Plasmid #65777), and *TubA1B* mEGFP/mEos 3.2 donor plasmid. Guide efficiency was determined by transfection with the mEGFP donor and Guide C was selected on the basis of the efficiency of mEGFP tagging in Hek293T cells. Guide C was used to generate clones in this study.

Suspension cells were transfected using the Neon Transfection System (Thermo Scientific). Cells were suspended at 1 × 10^6^ per reaction condition in Buffer R with 1 µg DNA before transfection in 10 µl transfection tips as described by the manufacturer (2x pulses, 20ms pulse width, 1450 V).

### Guide and Donor Vector Cloning

Guide sequences were generated using the MIT CRISPR database, and selected on the basis of inverse likelihood of off-target binding. Guide sequences were ordered as complementary oligos (Supplementary Table 1), suspended at 10 µM and annealed to form a phosphorylated oligo duplex by incubation at 37 °C in a thermal cycler (Sense Quest) with 2 µl 10x T4 DNA Ligase Buffer (NEB), 0.5 µL T4 PNK (NEB), and 15.5 µL H_2_O. The BPK150 guide plasmid was digested overnight with BsmBI (NEB) and treated with 0.5 µL T4 Calf Intestinal Phosphatase (NEB) to generate linearised and dephosphorylated vector backbones with overhangs complementary to the guide oligo duplexes. Finally the digested vector (2 µg per guide) and oligo (0.5 µM) duplexes were ligated at room temperature for 30 minutes with 1 µL 10x T4 DNA ligase buffer (NEB) and 0.5 µL T4 DNA Ligase (NEB). 2 µL of ligated vector were transformed into Alpha-Select Silver Efficiency *E. coli* cells (Bioline), by firstly incubating the DNA and bacteria on ice for 30 minutes, followed by 40 seconds of heat shock at 40 °C, and a further 2 minutes on ice. Cells were then suspended in 950 µL SOC medium (Thermo) and incubated on a rotary shaker for 1 hour at 37 °C before plating on agar plates with ampicillin at 100 µg/mL and incubated at 37 °C overnight. The following afternoon colonies were selected and grown overnight on a rotary shaker at 37 °C in liquid ampicillin containing LB Broth.

Colony PCR was performed to verify the correct insertion of guide oligo duplexes. 2 µL of guide plasmid bacterial solution from the overnight cultures was added to 500 nM of forward OS280 primer (5′-CAGGGTTATTGTCTCATGAGCGG-3′) and 500 nM of the reverse guide oligo used to generate the guide duplexes. 25 µL RedTaq ReadyMix PCR Reaction Mix (Sigma) and the necessary amount of dH_2_O was used in the PCR mix to yield a final reaction volume of 50 µL. Colony PCR was performed using a thermal cycler with a program of 1 minute denaturing at 94 °C, 2 minutes annealing at 54.5 °C and 3 minutes extension at 72 °C. The resulting PCR product was then run alongside a 1 kb ladder (NEB) through a 1.5% agarose gel with 3 µL ethidium bromide for 25 minutes at 120 V.

Donor templates were generated to introduce fluorescent tags by Gibson cloning. Homology arms, mEos 3.2, and mEGFP sequences were designed to include 20 base pair homologous overhangs for a four fragment assembly using the HiFi Assembly kit (NEB). DNA was ordered as gBlocks from Integrated DNA Technologies (IDT) (Supplementary Table 2). DNA Fragments were assembled into a pGem-T Easy Vector backbone (Promega) using a 1:1 vector to insert ratio and added to 10 µL HiFi master mix, and deionised H_2_O added to make up a total reaction volume of 12.5 µL. Linearised pGem-T Easy vector in the absence of inserts was used as a negative control. The assembled donor vector was chilled on ice for 10 minutes prior to transformation as described above and were plated on ampicillin agar plates.

### Western Blotting

Lysates were prepared from clonal populations of cells in a proteolysis inhibitor (Sigma) before solution in 2x reducing buffer. Western Blots were resolved by SDS-PAGE on 4–12% Bis/Tris gradient NuPage gels (Thermo Scientific). Proteins were transferred to polyvinylidene difluoride (PVDF; Bio-Rad) membranes using a Bio-Rad semi-dry Turbo transfer system. Membranes were blocked with 4% BSA in 0.1% Tween-20 Tris-buffered saline (TBST; 200 mM Trizma base, 1.37 M NaCl, 0.1% Tween-20) and probed with antibodies to α-tubulin (Sigma), GAPDH (loading control) (Abcam) and after antibody stripping with antibodies to eGFP (Sigma) and α-tubulin (Sigma). For quantification of TubA1B expression, wild type lysates (Hek293T, Hel 92.1.7, A549) were first probed with a TubA1B antibody (Abcam) and a β-actin loading control before stripping and re-probing with antibodies to α-tubulin (Sigma) and GAPDH (loading control). Values were normalized to loading controls, and then normalized to brightness to give relative intensity values. All primary antibodies were diluted in the blocking buffer shown above. Secondary incubations were performed in anti-mouse 680 and anti-rabbit 800 fluorescent antibodies (LiCor Instruments) for fluorescent detection using an Odyssey Fc (LiCor Instruments).

### Fluorescence Cell Sorting and Flow Cytometry

Fluorescence cell sorting was performed approximately one week after transfection using a BD FACSAria Fusion cell sorter with 100um nozzle at 20 psi. Gates were set on the brightest 1% of cells positive for fluorescence. Expression level as total cellular fluorescence of individual clones was determined with an Accuri C6 Flow Cytometer (BD Technologies).

### Super-resolution Imaging (dSTORM, PALM and SRRF)

Cells were seeded on glass-bottomed 35mm MatTek Dishes (MatTek Corporation) 24 hours prior to fixation. Hel 92.1.7 cells were treated with PMA (phorbol 12-myristate 13-acetate) (Sigma) and thrombopoeitin (TPO - gift from Ian Hitchcock) overnight to induce differentiation and spreading. Cells were washed twice with PBS before clearance with microtubule stabilising buffer (MTSB) and fixation with ice cold methanol at −20 °C for 3 minutes. Samples were rehydrated over 3 successive 5 minute washes with TBST before imaging in either PBS for PALM or blinking buffer (50 g/mL glucose oxidase, 1 g/mL catalase, and 100 mM mercaptoethylamine-HCL in PBS) for dSTORM.

dSTORM, PALM and SRRF imaging was performed using a Nikon N-STORM system on a Ti-E stand with Perfect Focus, an Andor iXon Ultra DU-897U EMCCD camera, and an Agilent MLC400 high power laser bed. Images were acquired through a 100 × 1.49 NA TIRF Objective lens and NIS Elements 4.2 software. Images were reconstructed using the ThunderSTORM^24^ plug-in in Fiji using a Maximum Likelihood and Integrated Gaussian PSF fitting or NanoJ-SRRF^25^ in Fiji. ThunderSTORM localization data was used to generate mean and mode xy data and localization counts (referred to as number of localizations in figures).

Full width half maximum (FWHM) was measured across microtubules by drawing a 5μm line on normalized Gaussian images derived from ThunderSTORM in Fiji. The resulting plot profile was then exported into Igor Pro (6.37) for multi-peak fitting, and the resulted FWHM were tabulated in Graph Pad PRISM. Fourier Ring Correlation analysis was performed using the FRC plug-in provided by BIOP within Fiji^26, 27^. For FRC analysis the Fixed 1/17 thresholding method was applied. For both FWHM and FIRE calculations, 3 independent images were used.

### Statistical Analysis

Statistical analysis was performed using Graph Pad PRISM 6. An *n* of 3 was acquired for both quantitative western blotting and image analysis. Significance was calculated using a 2-way ANOVA with multiple comparisons (Tukeys), with bars for each graph representing standard deviation of the mean.

## Electronic supplementary material

Video S1

Video S2

Supplementary information
