# Supplementary material for: CRISPR-Cas9 Mediated Labelling Allows for Single Molecule Imaging and Resolution
Source: Sci Rep. 2017 Aug 16;7:8450. doi: 10.1038/s41598-017-08493-x (PMC5559501; doi:10.1038/s41598-017-08493-x)
Supplement: Supplementary file 3 — Supplementary information [file 41598_2017_8493_MOESM3_ESM.pdf]

CRISPR-Cas9 Mediated Labelling Allows for Single Molecule Imaging and Resolution

Abdullah O. Khan[1], Victoria A. Simms[1], Jeremy A. Pike[2], *Steven G. Thomas[1,2], *Neil V. Morgan[1]

1. Institute of Cardiovascular Sciences, College of Medical and Dental Sciences, University of Birmingham, UK.
2. Centre of Membrane Proteins and Receptors (COMPARE), Universities of Birmingham and Nottingham, Midlands, UK.
* Joint corresponding authors

Supplementary Figure 1: Design of Homology Vector and Guides

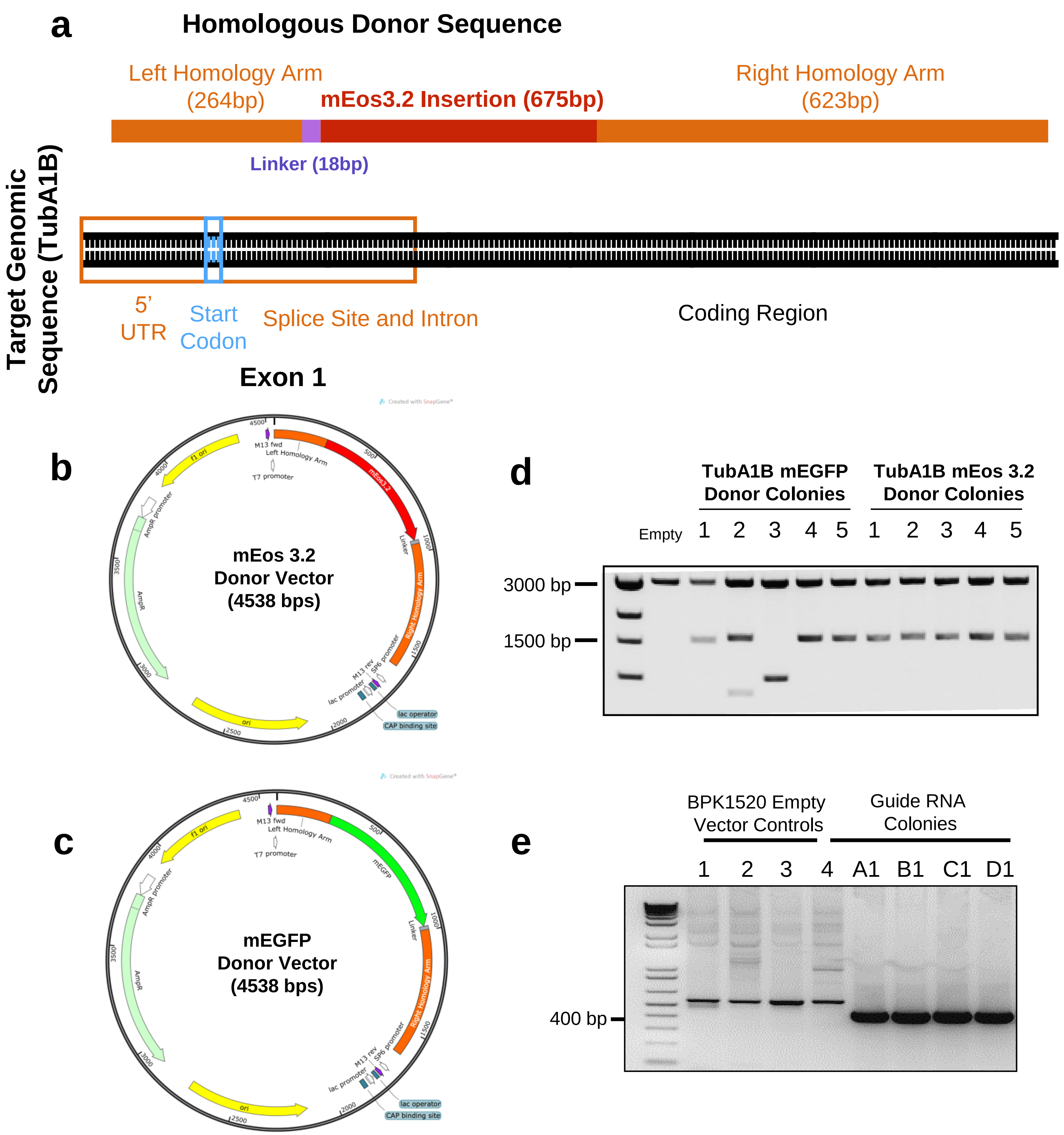

Supplementary Figure 1: Design and cloning of mEos 3.2 and mEGFP donors and *TubA1B* guide RNAs. (a) A donor sequence comprised of mEos 3.2 (675 bp) flanked by left (264 bp) and right (643 bp) homology arms was designed to target Exon 1 of the human *TubA1B* gene. (b) The donor sequence was subsequently cloned into a pGem-T easy vector. (c) Similarly an mEGFP donor vector was designed and cloned. The final vector design is comprised of the donor sequence and flanking regions integrated into a linearised pGem T-easy vector at a final size of 4538 base pairs. (d) After cloning the necessary sequences into the pGem T-easy backbone, a test digest was performed comparing the empty vector to colonies obtained after cloning. All colonies are approximately 1500 bp heavier when compared to the linearized control, indicating correct assembly the fragments. This was further confirmed by sequencing of the plasmid. (e) Cloning of the 4 targeting guide RNAs into the BPK 1520 vector was confirmed with a colony PCR which shows the presence of a 400 base pair band in vectors with the insert compared to control. This was further confirmed by sequencing of the vector.

Supplementary Figure 2: Fluorescent Cell Sorting

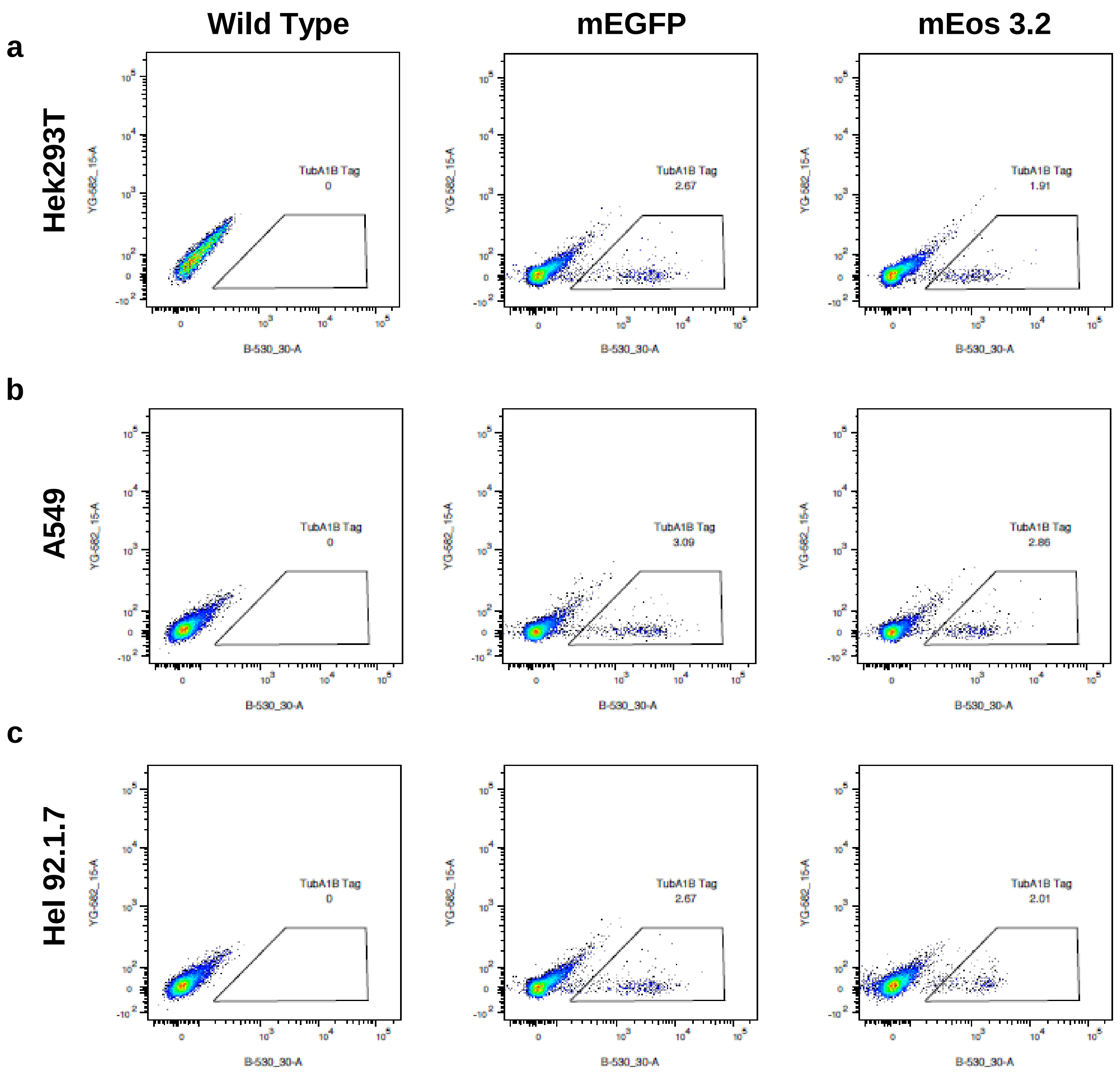

Supplementary Figure 2: Fluorescent single cell sorting of CRISPR-Cas9 tagged *TubA1B* with mEGFP and mEos 3.2 in (a) Hek293T, (b) A549, and (c) Hel 92.1.7. CRISPR treated cells are positive for green fluorescence which is lacking in wild type cells as indicated by control blots. For single cell sorting, gates were set to the top 1% of cells to ensure the selection of highly expressing clones.

Supplementary Figure 3: Clonal Verification

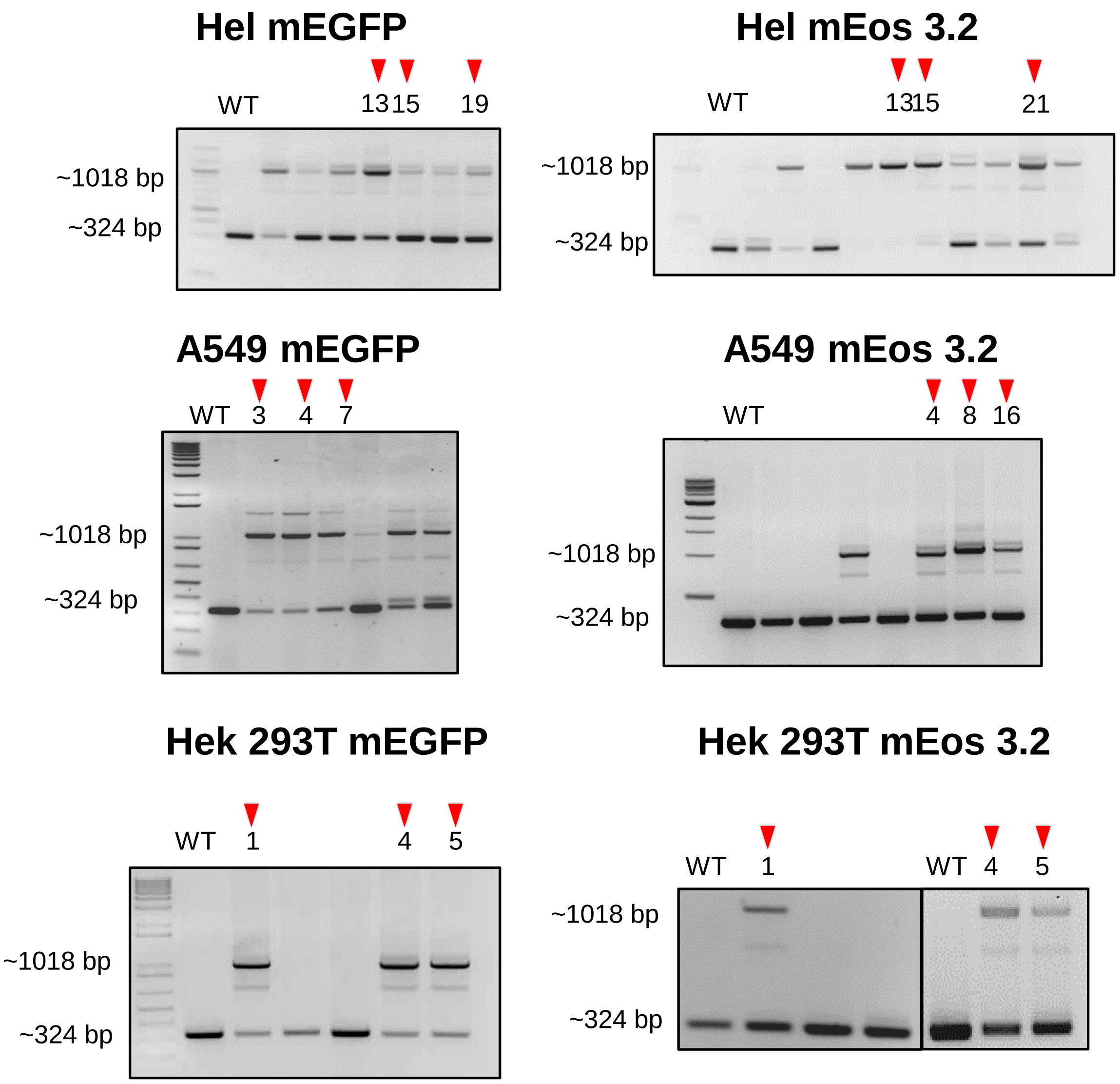

Supplementary Figure 3: Clonal verification of mEos 3.2 and mEGFP insertion into the N-terminal coding region of TubA1B gene. PCR amplification of a ~324 base pair region of Exon 1 of TubA1B allows for the detection of both mEGFP and mEos 3.2 insertions. Clones were selected on the presence of the appropriate insert and level of fluorescence for western blotting (indicated by red arrow). Un-labelled clones were not taken further for study.

Supplementary Figure 4: Full Page PALM and dSTORM Images

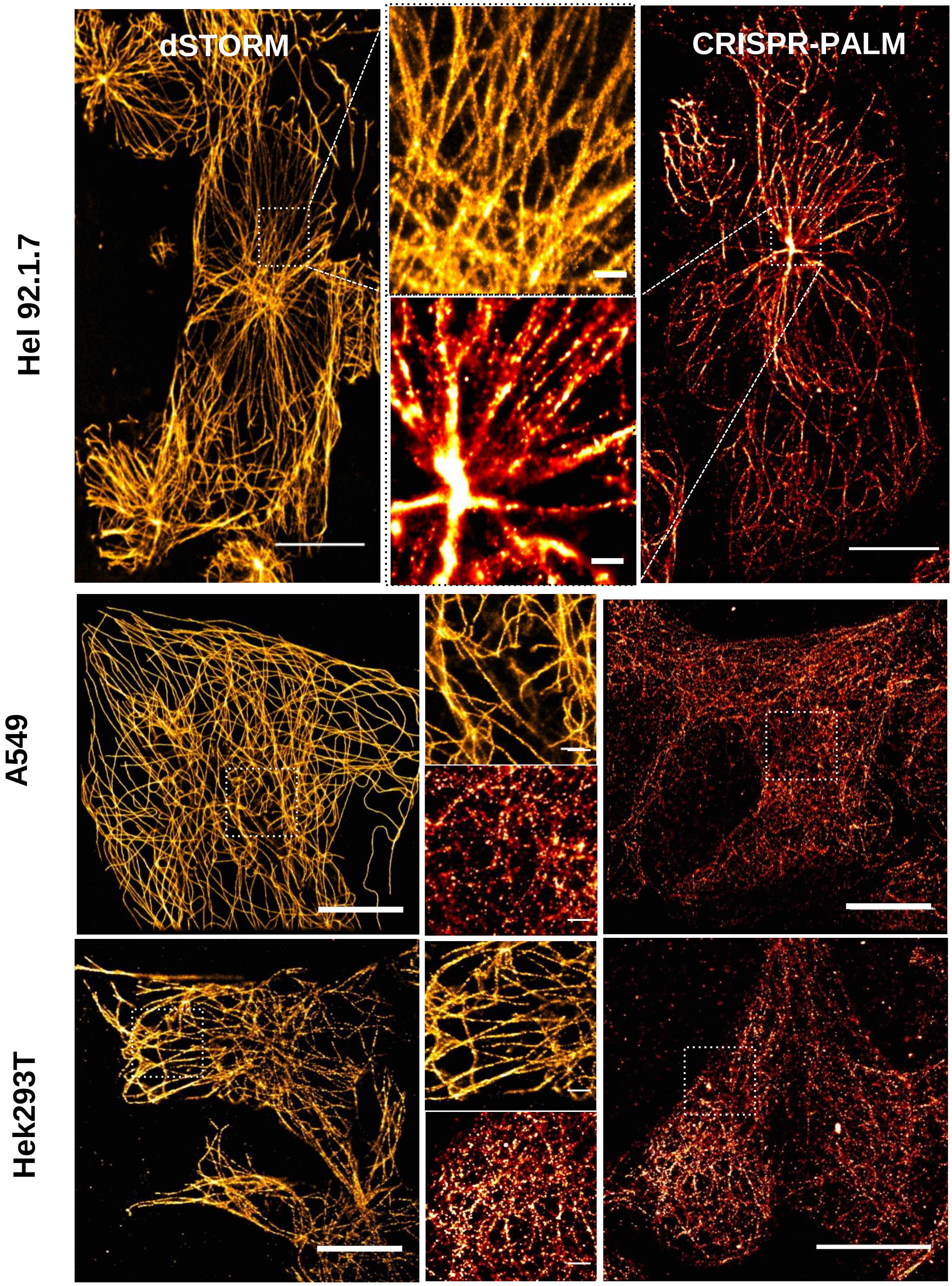

Supplementary Figure 4 Full sized overview of dSTORM and CRISPR-PALM images. While photoswitching of CRISPR tagged TuBA1B-mEos 3.2 allows for PALM imaging of A549 and Hek293T, spatial resolutions comparable to dSTORM are achievable in Hel 92.1.7 where expression of the knocked-in mEos 3.2 are highest.

Supplementary Figure 5: Confirmation of SRRF Super-Resolution

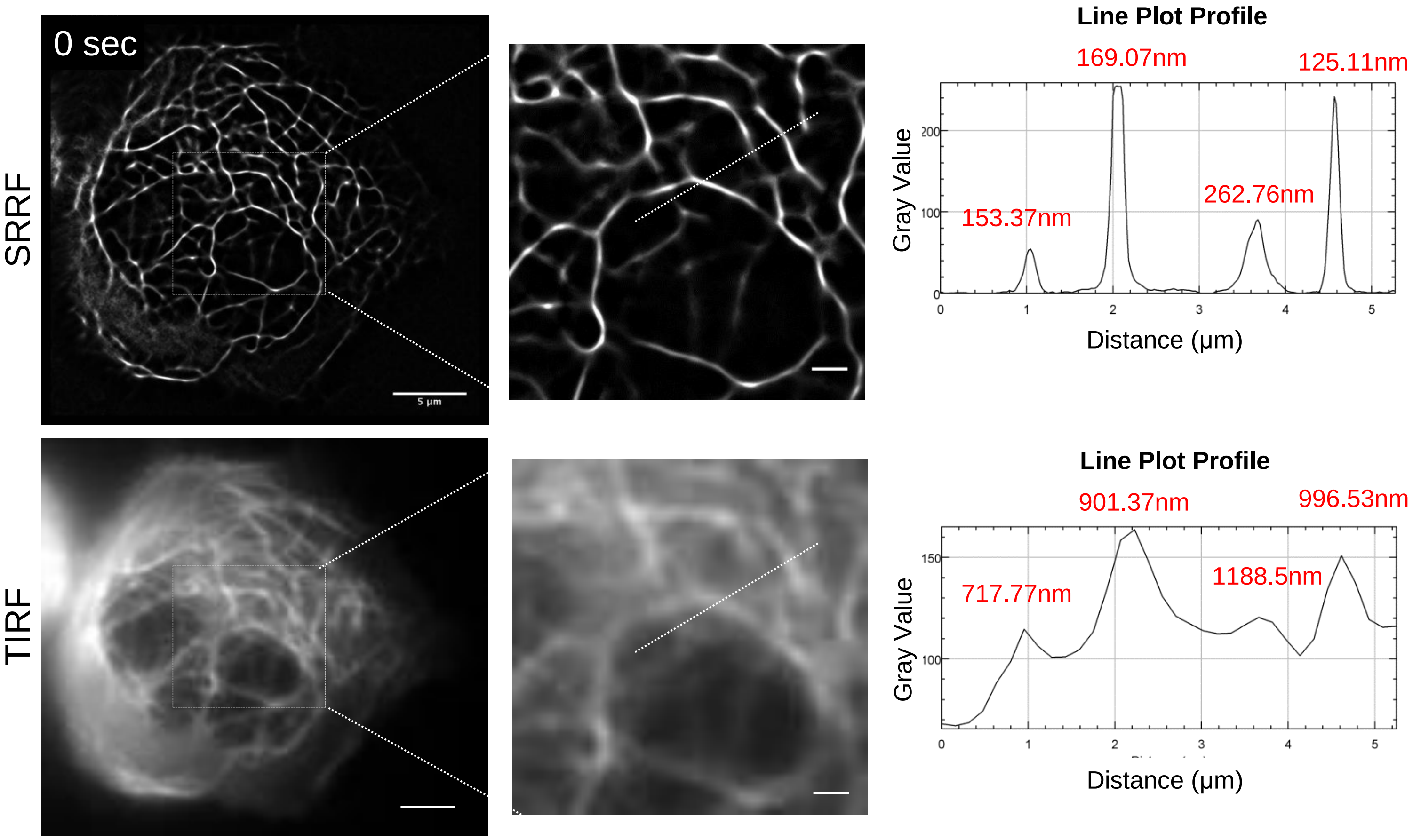

Supplementary Figure 5 SRRF imaging of CRISPR tagged mEos 3.2 allows for rapid, super-resolved live cell imaging using SRRF super resolution imaging. Improved resolution is observed as evidenced qualitatively by the cropped images of a Hel 9.2.17 cell, and quantitatively by the full width half maxima (FWHM) measured.

Supplementary Figure 6: *TubA1B* Expression Across Cell Lines

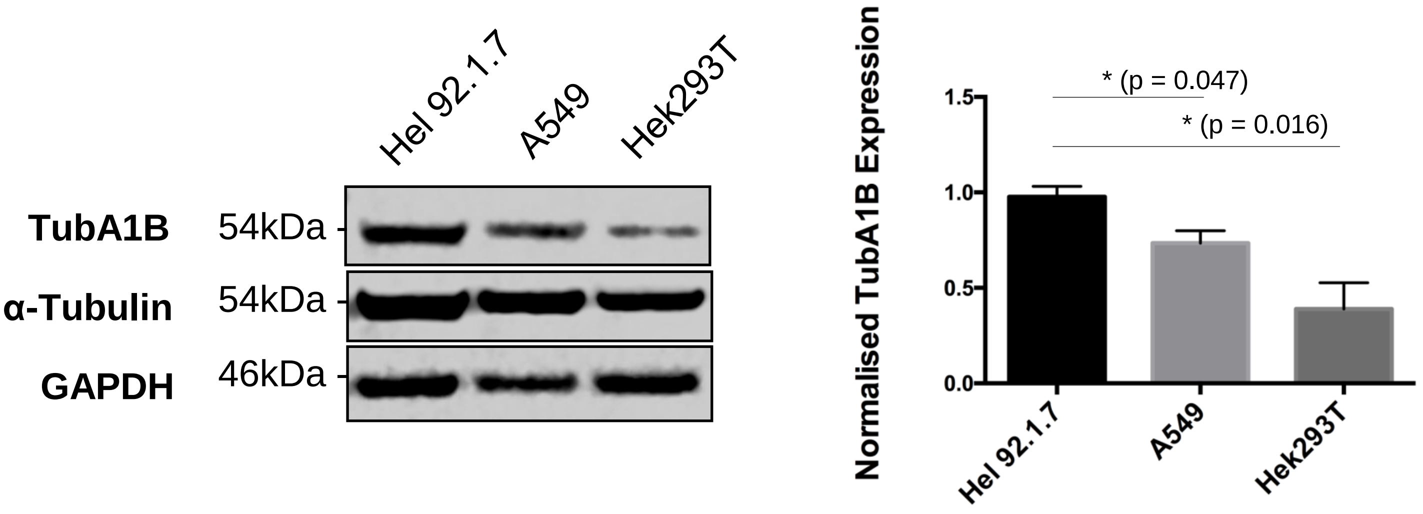

Supplementary Figure 6 Comparison of TubA1B Expression across three tested cell lines. Normalised *TubA1B* expression is highest in Hel 92.1.7 when compared to A549 (* p = 0.047) and Hek293T (* p = 0.016) cells (Multiple T-Test with significance determined by Holm-Sidak method). This difference in expression level is likely to be a factor in the different expression level of tagged tubulin observed.

Supplementary Figure 7: Full Sized Western Blots (Figure 1)

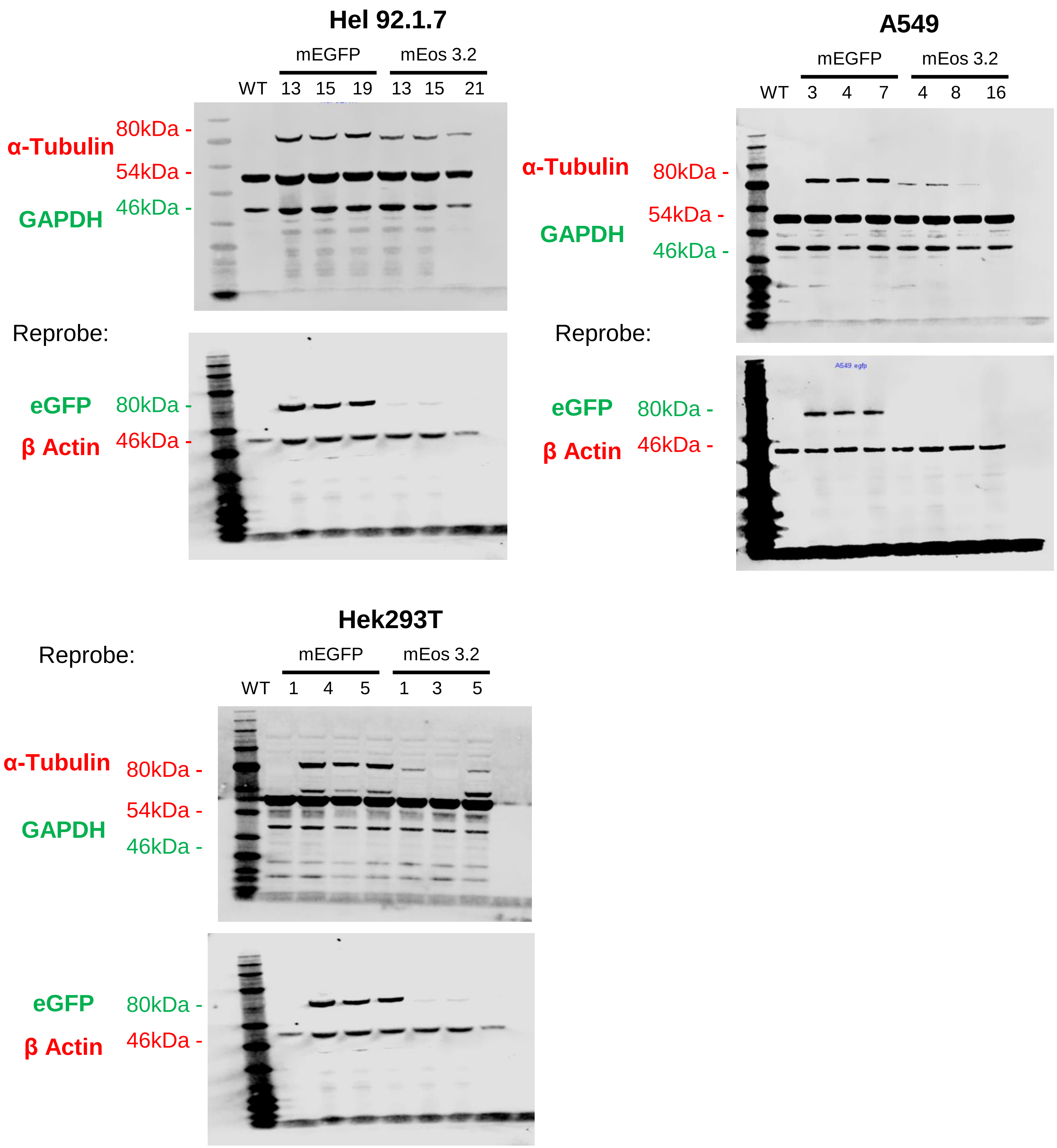

Supplementary Figure 7 Complete raw gels as presented in Figure 1 in the main manuscript. Gels were first probed with α-tubulin and GAPDH primary antibodies (and Odyssey fluorescent secondary antibodies anti-mouse (680 indicated in red) and anti-rabbit (800 indicated in green) respectively), then stripped and re-probed for eGFP (rabbit 800) and β Actin (mouse 680). All images acquired through the Odyssey LiCor Imaging system at identical exposure times (2 min 30 seconds), and presented here as acquired with no background subtraction.

Supplementary video legends

Supplementary video S1 – Averaged widefield timelapse recording of a Hel92.1.7 cell expressing TubA1B-mEos. Cells were recorded continuously at approx. 100 fps for 100 seconds.

Supplementary video S2 – SRRF reconstruction of the cell in video S1 showing live cell sub-second super-resolution imaging of microtubules in the CRISPR-mEos Hel cells.

Supplementary Table 1: Oligo Sequences

**TubA1B_gA_Top_**	CACCGCCTCGACTCTTAGCTTGTCG
**TubA1B_gA_Bottom_**	AAACCGACAAGCTAAGAGTCGAGGC
**TubA1B_gB_Top_**	CACCGCCCCGACAAGCTAAGAGTCG
**TubA1B_gB_Bottom_**	AAACCGACTCTTAGCTTGTCGGGGC
**TubA1B_gC_Top_**	CACCGACCTCGACTCTTAGCTTGTC
**TubA1B_gC_Bottom_**	AAACGACAAGCTAAGAGTCGAGGTC
**TubA1B_gD_Top_**	CACCGATTAGGAGGCGAAGGCGAC
**TubA1B_gD_Bottom_**	AAACGTCGCCTTCGCCTCCTAATC
**jPCR TubA1B Fwd Primer**	ACTCTTAGCTTGTCGGGGAC
**jPCR TubA1B Rev Primer**	CCCTTCCAGAGTCCGAGAAG

Supplementary Table 1: List of oligos ordered as guide RNAs and primers for genotyping PCRs (jPCRs).

Supplementary Table 2: gBlock Sequences for Donor Plasmid

**Left Homology Arm**	CATGGCGGCCGCGGGAATTCGATCGGAGTGGGCGCGCGGGGCCGGAGGA<br>GGGGCCAGCGACCGCGGCACCGCCTGTGCCCGCCCGCCCCTCCGCAGCC<br>GCTACTTAAGAGGCTCCAGCGCCGGCCCCGCCCTAGTGCGTTACTTATCTC<br>GACTCTTAGCTTGTCGGAGACGGTAACCGGTACCCGGTGTCTGCTCCTGTC<br>GCCTTCGCCTCCTAATCCCTAGCCACTATGGGAGTGAGTG
**mEos 3.2 Insert**	AGCCACTATGGGAGTGAGTGTGATTAAACCAGACATGAAGATCAAGCTGCG<br>TATGGAAGGCGCTGTAAATGGACACCCGTTCGCGATTGAAGGAGTTGGCCT<br>TGGGAAGCCTTTCGAGGGAAAACAGAGTATGGACCTTAAAGTCAAAGAAGG<br>CGGACCTCTGCCTTTCGCCTATGACATCTTGACAACTGTGTTCTGTTACGGC<br>AACAGGGTATTCGCCAAATACCCAGAAAATATAGTAGACTATTTCAAGCAGT<br>CGTTTCCTGAGGGCTACTCTTGGGAACGAAGCATGAATTACGAAGACGGGG<br>GCATTTGTAACGCGACAAACGACATAACCCTGGATGGTGACTGTTATATCTA<br>TGAAATTCGATTTGATGGTGTGAACTTTCCTGCCAATGGTCCAGTTATGCAG<br>AAGAGGACTGTGAAATGGGAGCCATCCACTGAGAAATTGTATGTGCGTGAT<br>GGAGTGCTGAAGGGTGATGTTAACATGGCTCTGTCGCTTGAAGGAGGTGGC<br>CATTACCGATGTGACTTCAAAACTACTTATAAAGCTAAGAAGGTTGTCCAGTT<br>GCCAGACTATCACTTTGTGGACCACCACATTGAGATTAAAAGCCACGACAAA<br>GATTACAGTAATGTTAATCTGCATGAGCACGCCGAAGCGCATTCTGAGCTGC<br>CGAGGCAGGCCAAGTCCGGACTCAGATCTCGAGTGAGTAAGC
**mEGFP Insert**	AGCCACTATGGGAATGGTGAGCAAGGGCGAGGAGCTGTTCACCGGGGTGG<br>TGCCCATCCTGGTCGAGCTGGACGGCGACGTAAACGGCCACAAGTTCAGC<br>GTGTCCGGCGAGGGCGAGGGCGATGCCACCTACGGCAAGCTGACCCTGAA<br>GTTCATCTGCACCACCGGCAAGCTGCCCGTGCCCTGGCCCACCCTCGTGAC<br>CACCCTGACCTACGGCGTGCAGTGCTTCAGCCGCTACCCCGACCACATGAA<br>GCAGCACGACTTCTTCAAGTCCGCCATGCCCGAAGGCTACGTCCAGGAGCG<br>CACCATCTTCTTCAAGGACGACGGCAACTACAAGACCCGCGCCGAGGTGAA<br>GTTCGAGGGCGACACCCTGGTGAACCGCATCGAGCTGAAGGGCATCGACT<br>TCAAGGAGGACGGCAACATCCTGGGGCACAAGCTGGAGTACAACTACAACA<br>GCCACAACGTCTATATCATGGCCGACAAGCAGAAGAACGGCATCAAGGTGA<br>ACTTCAAGATCCGCCACAACATCGAGGACGGCAGCGTGCAGCTCGCCGAC<br>CACTACCAGCAGAACACCCCCATCGGCGACGGCCCCGTGCTGCTGCCCGA<br>CAACCACTACCTGAGCACCCAGTCCAAGCTGAGCAAAGACCCCAACGAGAA<br>GCGCGATCACATGGTCCTGCTGGAGTTCGTGACCGCCGCCGGGATCACTC<br>TCGGCATGGACGAGCTGTACAAGTCCGGACTCAGATCTCGA*GTGAGTAAGC*<br>*CGT*
**Right Homology Arm**	*CAGATCTCGA*GTGAGTAAGCCGTGCGGCTCCCGGCTGCTTTCAGGGAAGC<br>AGGGAAAAGCGAGCCGGCGGGGCGCTGGGGCCCTGTATACAGCCGGGAA<br>GGGCTGGCCTCAGAGCCGTCCGTTTGGAGGGCGGAAAACGAGGCGAGAG<br>GCCAGGGCGGGAGTGGTGAGACCTCGGTGTGTGTAAATAGCGGGGGCCC<br>GGAAAGGTCGAGGGGCGCCAGGATTTCTTCTCGGACTCTGGAAGGGATGG<br>GGGGCTCGGGCTGCCCTCCGCCGTATCCGGAGCTCTCTTTTGTCGCGTAAC<br>TGTGTCCTGGGTGCGGTCCCTCGAGTCCCCGCAGTCCTTTCCAGCGCATGC<br>CCTTACTCCGCCTTGGGTGGACGCGCGCGCGGACTCTTCCAGCCCTCACTT<br>CCTCTTGAGCGCGAAAAGCGGGGGTGGGAAGCAGCTGGAGACAAAAGCGC<br>GCACGCGCGACCGTTACCTTCCCGCCGCTCCTGGGCGGGAAACCGCCACT<br>GCGCTTTGCGCATGCGCTCTGGGTCGCGACGCGACTAGGGCTACAGGGCG<br>TGTCTCCTGTTAACCTGAGTGTTCTTTTTTTGATGAAAGCAATAAGAGGACTG<br>CGNGAAGAGCTCCCTGTCAATGTACCGCTCTACAATCACTAGTGAATTCGC<br>GGC

Supplementary Table 2: List of gBlock sequences ordered for cloning into *TubA1B* donor plasmids.
